# The impact of DRG payment reform on inpatient costs for different surgery types: an empirical analysis based on Chinese tertiary hospitals

**DOI:** 10.3389/fpubh.2025.1563204

**Published:** 2025-06-03

**Authors:** Mingwei Luo, Hongying Li, Rongyue Li, Yugao Wu, Yuping Lan, Shiwei Xie

**Affiliations:** ^1^Department of Medical Records Statistics, Panzhihua Central Hospital, Panzhihua, Sichuan, China; ^2^Department of Medical Records Statistics, Sichuan Provincial People's Hospital, Chengdu, Sichuan, China; ^3^Department of Orthopaedics, Panzhihua Central Hospital, Panzhihua, Sichuan, China

**Keywords:** DRG payment reform, surgery types, inpatient costs, Chinese tertiary hospitals, multivariate regression analysis

## Abstract

**Background:**

To evaluate the impact of DRG (Diagnosis-Related Group) payment reform on inpatient costs for four major types of surgery at a tertiary hospital in China, with a focus on its implementation in general surgery, cardiothoracic surgery, neurosurgery, and urology.

**Methods:**

Based on inpatient data from 2019 to 2023, the study employed Propensity Score Matching (PSM) and Difference-in-Differences (DiD) models to compare inpatient cost differences between the DRG payment group and the non-DRG payment group. Concentration indices were used to assess the consistency of inpatient cost distribution.

**Results:**

The DRG payment reform significantly reduced the total inpatient costs for surgeries in general surgery, cardiothoracic surgery, neurosurgery, and urology, particularly in terms of drug and material expenses. Additionally, the inpatient cost distribution in the DRG payment group became more concentrated, indicating a significant reduction in the proportion of high-cost cases.

**Conclusion:**

The DRG payment reform effectively controlled inpatient costs in Chinese tertiary hospitals, particularly for complex surgical procedures, and improved the efficiency of healthcare resource utilization. This study provides empirical support for the further implementation of DRG payment reform and offers policy recommendations for optimizing China’s healthcare payment system.

## Background

The DRG (Diagnosis-Related Group) payment system was developed by Yale University in the United States during the 1970s, with the aim of controlling healthcare costs and improving hospital service efficiency by categorizing and charging based on disease types (1). Initially developed as a tool for hospital management, DRGs became the basis of the inpatient prospective payment system that Medicare implemented in 1983 (2). Since its introduction, this system has been gradually adopted in several developed countries, including Germany, Australia, and France. The core concept of the DRG payment system is to charge based on disease types rather than individual services, thereby helping healthcare institutions control costs while providing high-quality care, and optimizing the allocation of medical resources. In the United States, the DRG payment system was first applied to the Medicare payment model in 1983 and quickly spread nationwide (2). It is generally expected that the implementation of DRG payment systems may help reduce the length of hospital stay and inpatient costs through standardized service delivery. However, whether such changes affect the quality of care remains a subject of ongoing evaluation. In Europe, Germany fully implemented the DRG payment system in 2003 and has continuously optimized it to better manage the costs of high-complexity cases (3). Australia introduced the DRG payment system in the early 2000s and successfully expanded it to emergency and outpatient services, demonstrating its broad adaptability across different healthcare services (4).

In recent years, the rapid growth of healthcare costs in China has placed significant economic pressure on both the government and patients. Particularly in terms of inpatient costs, the traditional Fee-for-Service (FFS) model has led to the overuse of medical resources and uncontrolled expenses (5). Consequently, controlling healthcare costs and improving the efficiency of medical services have become central tasks in China’s healthcare reform. To address this challenge, China began piloting DRG payment reform in selected cities starting in 2012 (6). The reform aims to guide hospitals in the efficient use of resources, reduce unnecessary medical expenditures through well-designed policies, and gradually expand nationwide. Preliminary studies have shown that DRG payment reform in China has the potential to control healthcare costs and regulate medical service practices (7). However, compared to developed countries, China faces several unique challenges in implementing DRG payment, including the development of DRG classification standards tailored to the Chinese context and the adaptability of hospitals to the new payment model.

Tertiary general hospital are the core strength of China’s healthcare system, undertaking the diagnosis and treatment of complex diseases, as well as medical education and research tasks (8). These hospitals not only possess advanced medical equipment and technologies but also concentrate a large number of highly qualified medical professionals. Consequently, the clinical practices and management experiences of tertiary hospitals significantly influence the national standards for medical services and the formulation of policies. In-depth studies of these hospitals help to understand the operational mechanisms of China’s healthcare system and provide empirical support for policy-making.

In this context, the DRG payment system, as a new form of healthcare payment, is highly anticipated to control medical costs and improve the quality of healthcare services (9). The hospital selected for this study, Panzhihua Central Hospital, is a representative tertiary comprehensive hospital in Southwest China, which implemented DRG payment reform relatively early (in 2019) and thus serves as a benchmark for assessing regional implementation effects. In the sample hospital, the DRG payment reform was implemented in 2019, initially covering four major surgical specialties: general surgery, cardiothoracic surgery, neurosurgery, and urology. These surgical categories were selected based on their high prevalence and significant contribution to inpatient costs. However, the differences in resource utilization, complexity, and clinical pathways among different types of surgeries may lead to significant variations in the impact of DRG payment across various surgical procedures. Therefore, this study aims to explore the impact of DRG payment reform on inpatient costs for different types of surgeries. By analyzing changes in inpatient costs across different surgical types before and after the implementation of DRG payment reform in a tertiary general hospital, this study evaluates the effectiveness of DRG payment in controlling medical expenses, optimizing hospital resource allocation, and improving consistency in healthcare service delivery. While the findings are based on data from a single institution and may have limitations in generalizability, they offer valuable insights into the practical effects of DRG reform and provide preliminary empirical evidence to inform policy refinement and support the sustainable development of China’s healthcare system.

## Methods

### Study design

The study utilized a comparative design between the DRG payment group and the non-DRG payment group to assess the impact of DRG payment reform on inpatient costs. Specifically, surgical patients not involved in DRG payment were categorized into the non-DRG group, while those involved were categorized into the DRG group. The study utilized a comparative design between the DRG payment group and the non-DRG payment group to assess the impact of DRG payment reform on inpatient costs. In the sample hospital, DRG payment reform was implemented in 2019, targeting four major surgical specialties: general surgery, cardiothoracic surgery, neurosurgery, and urology. Specifically, surgical patients not involved in DRG payment during the initial implementation phase were categorized into the non-DRG group, while those covered under the reform were categorized into the DRG group. By comparing inpatient costs between these two groups, the study aimed to evaluate the effectiveness of DRG payment in controlling costs. To reduce confounding bias due to differences in patient characteristics, the study employed the Propensity Score Matching (PSM) method. The logistic regression model was expanded to include major comorbidities in a more detailed manner. Specifically, cardiovascular diseases were disaggregated into hypertension, coronary artery disease, and heart failure. Other comorbidities included diabetes and chronic respiratory diseases such as chronic obstructive pulmonary disease (COPD). This inclusion aimed to better control for potential confounders that could affect inpatient costs. Propensity scores were calculated based on variables such as age, gender, Charlson Comorbidity Index (CCI), and the individual comorbidities listed above. This economic analysis was conducted from the hospital/provider perspective, focusing on direct inpatient costs incurred by the hospital, including drug and material expenses, hospitalization days, and surgical resource use.

The analysis of cost differences employed the standard deviation (SD) to measure the dispersion of inpatient costs. A smaller SD indicates a more concentrated cost distribution, suggesting that DRG payment may be effective in controlling costs. The Double-Difference Value was used to compare the differences in cost changes between the DRG group and the non-DRG group. By calculating the cost changes in different years for both groups, the study assessed the impact of DRG payment reform. The Concentration Index was used to measure the distribution of inpatient costs across different patient groups, with a Concentration Index close to 0 indicating a more uniform distribution of costs. To further assess the balance between treatment and control groups, a two-sample t-test was conducted to compare key characteristics (e.g., age, gender, CCI) before and after matching. This test provided *p*-values to evaluate whether there were significant differences between the groups, thereby supporting the claim of relative balance.

The data analysis in this study was conducted using Stata SE 15.0 software. A linear regression model was used to analyze the relationship between inpatient costs and DRG payment, controlling for the influence of potential confounding factors. A Logistic regression model was employed to calculate propensity scores and perform matching. The Difference-in-Differences (DiD) model was used to assess the causal impact of DRG payment on changes in inpatient costs. The study analyzed the impact of DRG payment on cost dispersion by calculating the standard deviation (SD) for the DRG group and the non-DRG group across different types of surgeries. By calculating the interquartile range (IQR) of cost data, the study further assessed the concentration and distribution characteristics of costs; a smaller IQR indicates higher cost concentration. By analyzing the changes in the Concentration Index for different types of surgeries across different years, the study evaluated the impact of DRG payment on cost equity and consistency.

**Figure 1:**
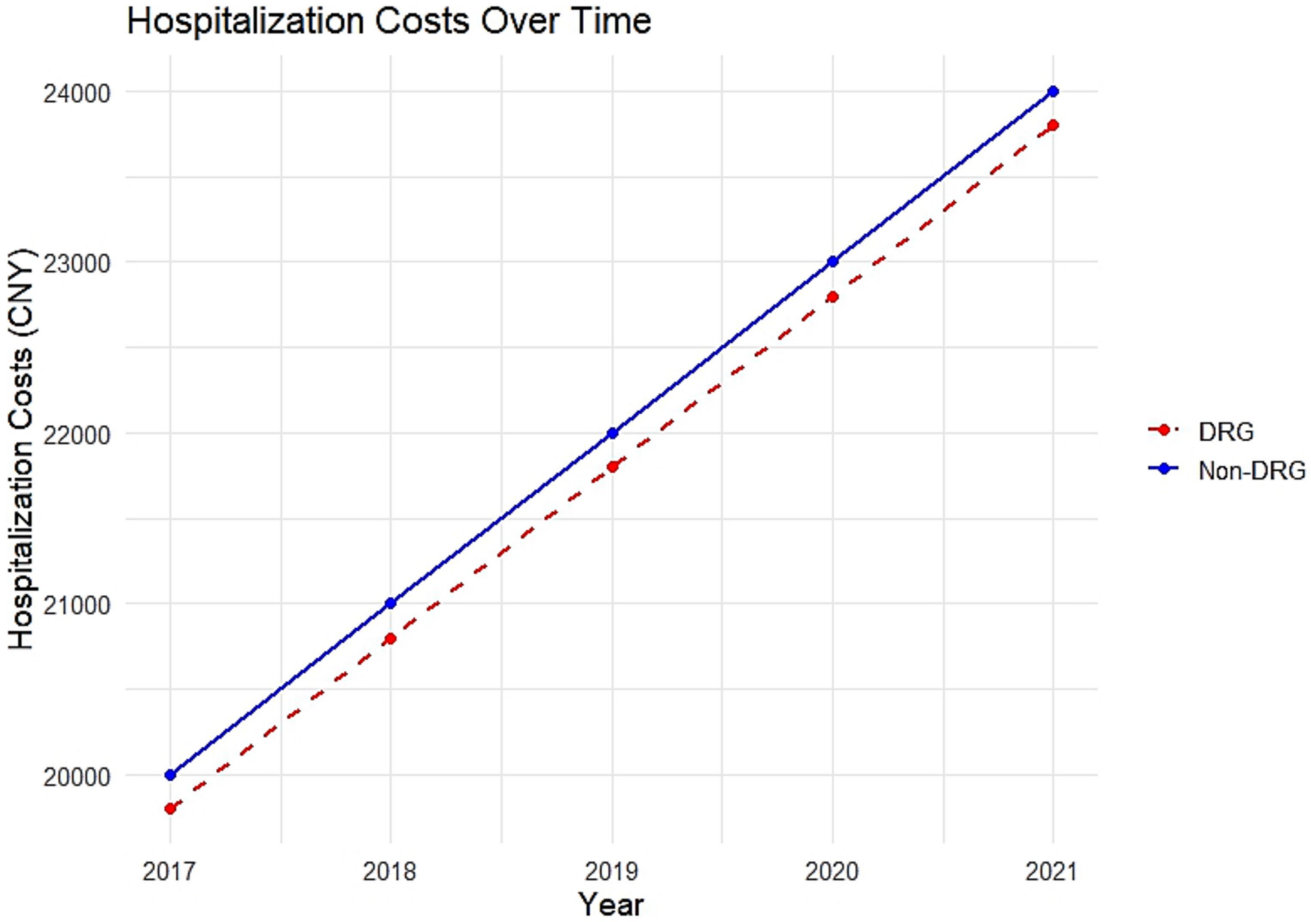
Parallel trend test for DRG and Non-DRG groups. This figure illustrates the results of the parallel trend hypothesis test, where the intervention and control group were parallel groups, satisfying the parallel trend hypothesis of the double differential method.

To further validate the Difference-in-Differences (DiD) method, a parallel trend test was conducted. This test aimed to confirm that the trends in inpatient costs for the DRG group and the non-DRG group were parallel before the implementation of the DRG payment reform. The results of this test, including a graphical analysis, are provided to demonstrate that the parallel trend assumption holds. The results of the parallel trend hypothesis test, as shown in Figure 1, before the DRG reform, the intervention and control groups were parallel, thus satisfying the parallel trend hypothesis of the dual differential method.

### Data source and sample selection

This study is based on inpatient data from a tertiary comprehensive hospital in China, covering patients who underwent general surgery, cardiothoracic surgery, neurosurgery, and urology surgery between 2019 and 2023. The sample hospital began implementing DRG payment reform in January 2019. To ensure the accuracy and representativeness of the study results, the sample selection focused on four common types of surgeries (Supplementary Table 1), with patients aged 18 and above, excluding pediatric patients to avoid differences in surgical complexity and costs. Patients were grouped according to the Charlson Comorbidity Index (CCI), with CCI ≤ 1 classified as the low comorbidity group and CCI > 1 as the high comorbidity group, thereby controlling for the severity of the patient’s condition in the analysis. Although data from a single tertiary hospital ensures detailed and internally valid findings, the lack of data from secondary hospitals, rural healthcare facilities, and specialty hospitals could potentially limit the generalizability of the conclusions.

### Data processing and propensity score matching

To reduce confounding bias due to differences in patient characteristics, this study employed the Propensity Score Matching (PSM) method (10). First, a Logistic regression model was used to calculate the propensity score for each patient, with matching variables including age, gender, Charlson Comorbidity Index (CCI), type of surgery, and year of surgery. These variables were used to estimate the probability of each patient receiving DRG payment reform. Next, 1: 1 Nearest Neighbor Matching without replacement and a caliper of 0.05 were applied to ensure matching precision and accuracy. After matching, standardized differences and t-tests were used to assess the balance of matching variables, with standardized differences less than 0.1 indicating good balance between variables. Additionally, a Love plot was drawn to display the distribution of variables before and after matching, further validating the matching effectiveness.

### Statistical model

To assess the causal impact of DRG payment reform on inpatient costs, the study employed a Difference-in-Differences (DiD) model (11). This model compares the changes in costs between the DRG payment group and the non-DRG payment group before and after the DRG payment reform, estimating the net effect of the reform. The specific setup of the DiD model is as follows:


$$Y_{\mathrm{it}}=\alpha +\beta_{1}\times {\text{Post}}_{t}+\beta_{2}\times {\mathrm{DRG}}_{i}+\beta_{3}\times ({\text{Post}}_{t}\times {\mathrm{DRG}}_{i})+X_{\mathrm{it}}\gamma +\in_{\mathrm{it}}$$

Here, Y_it_ represents the inpatient costs for patient i at time t; Post_t_ is a time dummy variable (indicating the period of DRG payment); DRG_i_ is a group dummy variable (DRG payment group versus non-DRG payment group); X_it_ represents control variables, including patient age, gender, CCI; and ϵ_it_ is the random error term. The coefficient of the interaction term (Post_t_ × DRG_i_), β_3_, captures the average treatment effect of the DRG payment reform.

To ensure the robustness of the model, a Placebo Test was conducted, where a “dummy” reform point was randomly set in years when the DRG payment reform was not implemented, to check for any significant changes in costs and validate the model’s robustness. A parallel trend test was also performed using pre-reform data to confirm whether the inpatient cost trends of the DRG payment group and the non-DRG payment group were parallel, thereby ensuring the validity of the DiD model assumption. To further verify the robustness of the model results, sensitivity analysis was conducted by altering the matching methods (such as adjusting the caliper value or using different matching algorithms) and the set of control variables (12). The study also analyzed the consistency of inpatient cost distribution using the Concentration Index to assess the impact of DRG payment on cost concentration. Changes in the Concentration Index were subjected to significance analysis using the Kruskal-Wallis test, to further evaluate the impact of DRG payment reform on cost equity.

### Cost standardization across years

To ensure comparability of hospitalization cost data across multiple years, all inpatient expenses were converted to constant 2019 values. This adjustment was based on the annual Consumer Price Index (CPI) for China, as published by the National Bureau of Statistics. Year-specific costs were adjusted using the formula:


$$\text{Adjusted Cost}=\text{Original Cost}\times ({\mathrm{CPI}}_{\left(2019\right)}/{\mathrm{CPI}}_{\left(x\right)})$$

where CPI₍x₎ represents the cumulative CPI for year x. The CPI values used were: 100.0 (2019, reference year), 102.5 (2020), 103.4 (2021), 105.5 (2022), and 106.6 (2023). All reported standard deviations and difference-in-difference values in Table 1 reflect inflation-adjusted figures expressed in 2019 CNY. In addition, to improve the accessibility of findings for international readers, we reported approximate USD equivalents for all monetary values based on the official average exchange rate in 2019 (1 USD ≈ 6.8985 CNY, as reported by the National Bureau of Statistics). All major cost figures are presented in both CNY and USD in Supplementary Table 3.

**Table 1:** The SD and double difference value of hospitalization expenses of patients (CNY).

Surgical department	Year	Control group (SD, CNY)	Control group change	Intervention group (SD, CNY)	Intervention group change	Double-difference value (CNY)
Cardiothoracic surgery	2019	27382.24		23329.7		
	2020	30068.47	3354.09	24671.74	1911.05	−1443.03
	2021	28428.84	−1377.91	28939.98	4482.99	5860.9
	2022	18304.61	−9558.35	16408.83	−11955.09	−2396.74
	2023	19106.8	991.08	16831.15	591.65	−399.43
General surgery	2019	15449.42		14510.53		
	2020	16635.76	1563.16	14524.22	367.61	−1195.55
	2021	15475.21	−1015.74	12740.71	−1657.11	−641.36
	2022	13889.64	−1277.54	11765.21	−721.88	555.65
	2023	12844.42	−901.9	12174.25	530.44	1432.34
Neurosurgery	2019	29304.24		25958.7		
	2020	44071.33	15481.82	39138.27	13812.71	−1669.11
	2021	43008.52	−679.2	42388.52	3590.91	4270.11
	2022	38285.93	−3866.5	38718.64	−2826.12	1040.38
	2023	30314.49	−7576.36	30432.05	−7887.05	−310.68
Urology	2019	11404.54		9746.49		
	2020	10826.37	−300.01	11483.32	1974.54	2274.55
	2021	12759.54	2027.4	10338.63	−1044.73	−3072.14
	2022	9983.12	−2522.44	8858.97	−1273.88	1248.56
	2023	10864.45	984.34	12540.37	3772.81	2788.48

## Results

### Description of sample characteristics

A total of 23,421 general surgery patients, 5,980 cardiothoracic surgery patients, 6,202 neurosurgery patients, and 9,629 urology surgery patients were included in this study (Table 2). There are basic demographic and clinical characteristics presented for patients in both the DRG and non-DRG payment groups, including age, gender, and Charlson Comorbidity Index (CCI). The table also includes the sample sizes (N values) for both groups. A two-sample t-test was conducted to confirm the balance of characteristics between the groups, and the standardized differences after propensity score matching were all less than 0.1, indicating good balance between the two groups. It also includes the *p*-values from the two-sample t-tests conducted for each characteristic, demonstrating that there were no statistically significant differences (*p* > 0.05) between the treatment and control groups after matching, confirming the relative balance achieved. After matching, the distribution of these characteristics between the DRG group and the non-DRG group became more consistent, thereby enhancing the credibility of the comparison results.

**Table 2:** Characteristics of samples.

Characteristic	Treatment group (*N*/%)	Control group (*N*/%)	Standardized difference (before matching)	Standardized difference (after matching)
Age (Mean ± SD)	52.17 ± 16.30	48.95 ± 16.63	0.15	0.02
Gender—Male (%)	9,794 (41.82%)	11,318 (44.86%)	0.10	0.01
Gender—Female (%)	13,627 (58.18%)	13,909 (55.14%)	0.10	0.01
CCI = 0 (%)	19,419 (82.91%)	21,136 (83.78%)	0.05	0.00
CCI = 1 (%)	3,544 (15.13%)	3,672 (14.56%)	0.04	0.01
CCI ≥ 2 (%)	458 (1.96%)	419 (1.66%)	0.03	0.02
Total *N*	23,421	25,227		

To assess the quality of the matching process, a Love plot was generated (see Supplementary Figure 1), which showed that all standardized mean differences were reduced to below 0.1 after matching. A paired t-test confirmed the improvement in covariate balance, with the mean standardized difference decreasing from 0.074 (SD = 0.050) to 0.012 (SD = 0.008), yielding a t-statistic of 2.878 and a *p*-value of 0.045.

### Analysis of inpatient cost differences

The composition of inpatient costs for DRG and non-DRG payment groups across four major surgeries is analyzed (Table 3). Overall, the total inpatient costs in the DRG payment group were lower than those in the non-DRG payment group across all surgery types, with particularly significant differences in drug and material costs. For example, in general surgery, drug costs accounted for 22.37% of the total costs in the DRG payment group, and material costs accounted for 20.65%, compared to 22.67% and 21.27% in the non-DRG payment group, respectively. Similar differences in cost composition were observed in cardiothoracic, neurosurgery, and urology surgeries, indicating that the DRG payment system has a clear advantage in cost control, especially in controlling drug and material costs. An additional heterogeneity analysis was conducted to assess the differential effects of DRG payment reform across the four types of surgery: general surgery, cardiothoracic surgery, neurosurgery, and urology. The analysis revealed that the impact of DRG payment reform varied significantly by surgical type. For example, the reduction in drug and material expenses was more pronounced in cardiothoracic surgery and neurosurgery, while general surgery and urology showed relatively moderate changes.

**Table 3:** Composition of hospitalization expenses of patients (CNY).

Surgical department	Drug expenditure				Material expenditure				Other expenditure			
	Pre-DRG	Post-DRG	*Z*	*p*	Pre-DRG	Post-DRG	*Z*	*p*	Pre-DRG	Post-DRG	*Z*	*p*
Cardiothoracic Surgery	5148.76 (23.84%)	4168.06 (23.42%)	−9.689	<0.001	6608.65 (30.59%)	5415.15 (30.43%)	−12.304	<0.001	9843.58 (45.57%)	8211.28 (46.15%)	−10.086	<0.001
General Surgery	3022.03 (22.67%)	2682.27 (22.37%)	−9.633	<0.001	2835.71 (21.27%)	2475.30 (20.65%)	−8.19	<0.001	7472.35 (56.06%)	6831.78 (56.98%)	−8.152	<0.001
Neurosurgery	5310.27 (18.56%)	4233.69 (17.54%)	−7.766	<0.001	9070.75 (31.71%)	8225.15 (34.08%)	−8.533	<0.001	14224.37 (49.73%)	11677.05 (48.38%)	−9.887	<0.001
Urology	2563.13 (22.61%)	2393.78 (22.67%)	−2.611	0.009	2399.06 (21.16%)	2,033.17 (19.26%)	−6.485	<0.001	6373.25 (56.22%)	6131.28 (58.07%)	−2.787	0.005

### Analysis of inpatient cost trends

**Figure 2:**
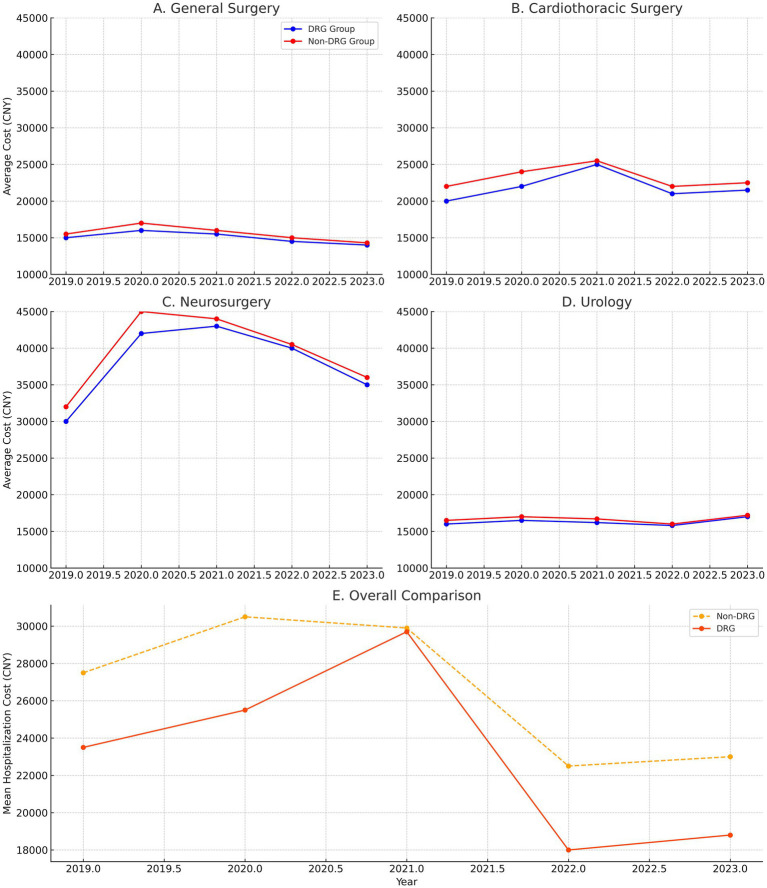
Difference-in-differences (DiD) analysis of hospitalization costs trends. **(A)** General surgery: The DRG payment group exhibits a steady decrease in hospitalization costs over time, particularly from 2020 to 2023. In contrast, the non-DRG group shows a more fluctuating and rising trend in costs. **(B)** Cardiothoracic surgery: Although there was some cost fluctuation in 2021, the overall trend in the DRG payment group demonstrates better cost control, particularly after 2021, with a more stable trend compared to the non-DRG group. **(C)** Neurosurgery: The DRG payment group shows a significant reduction in costs after 2021, with a marked decrease in hospitalization expenses from 2021 to 2023. This trend highlights the effectiveness of DRG in controlling costs for complex surgeries. **(D)** Urology: The DRG payment group shows a decreasing trend in costs year by year, with the most significant drop occurring in 2022, indicating the reform’s effectiveness in controlling expenses for this surgery type. **(E)** Overall comparison: The comparison of trends across all four surgery types (general surgery, cardiothoracic surgery, neurosurgery, and urology) demonstrates that, while the DRG payment reform resulted in cost reductions across all surgical types, the magnitude and consistency of these reductions varied. The DRG groups for most surgeries showed a steady decline in costs, while the non-DRG groups exhibited more variability and overall higher costs.

Table 1 shows the trends in hospital costs, including the standard deviation (SD) for the control and intervention groups. However, a smaller SD does not necessarily mean that DRG payments effectively control costs; it only indicates variability of reduced variability. In general surgery, the inpatient costs in the DRG payment group showed a decreasing trend year by year, with the double-difference value reaching 1432.34 (207.63 USD) yuan in 2023 (*p* < 0.01), indicating the DRG payment system’s effective cost control. In cardiothoracic surgery, although there was a cost increase in 2021, the overall trend showed that the DRG payment group had better cost control than the non-DRG payment group. Particularly in 2023, the double-difference value further confirmed the DRG payment’s effective suppression of inpatient cost growth. In neurosurgery, after 2021, the cost control in the DRG payment group significantly outperformed the non-DRG payment group, with a double-difference value of −310.68 (−45.04 USD) yuan in 2023 (*p* < 0.05), indicating the stability and long-term effectiveness of the DRG payment system in highly complex surgeries (Supplementary Table 4). In urology surgery, the inpatient costs in the DRG payment group showed a decreasing trend year by year, reaching a minimum in 2022. The double-difference value in 2023 significantly indicated the DRG payment system’s effective cost control in urology surgeries. Figure 2 (DID) plot to visualize the trends and changes in hospital costs over time between the intervention and control groups, providing a more intuitive understanding of the impact of the DRG payment reform.

### Concentration index analysis

To assess the impact of DRG payment reform on the consistency of inpatient cost distribution, changes in the Concentration Index of inpatient costs across different types of surgeries from 2019 to 2023 are presented (Table 4). The results showed that the Concentration Index for general surgery increased annually, rising from 0.46 in 2019 to 0.50 in 2023, indicating that the cost distribution became more concentrated and that the DRG payment system effectively reduced the proportion of high-cost cases. The Concentration Index for cardiothoracic surgery gradually increased from 0.53 in 2019 to 0.57 in 2023, reflecting an improvement in cost concentration after the reform, particularly in managing costs for complex surgeries. The Concentration Index for neurosurgery rose from 0.57 in 2019 to 0.59 in 2023. Although the change was modest, it reflects improved consistency and concentration in cost control. The Concentration Index for urology surgery significantly increased from 0.43 in 2019 to 0.52 in 2023, indicating that the DRG payment system has achieved notable success in controlling costs and improving distribution consistency in urology surgeries.

**Table 4:** The concentration index of hospitalization expenses of patients.

Year	Cardiothoracic surgery	General surgery	Neurosurgery	Urology
2019	0.53^***^	0.46^**^	0.57^***^	0.43^*^
2020	0.54^***^	0.46^**^	0.59^***^	0.42^*^
2021	0.58^***^	0.49^**^	0.57^***^	0.43^*^
2022	0.56^***^	0.49^**^	0.58^***^	0.45^*^
2023	0.57^***^	0.50^**^	0.59^***^	0.52^*^

The *p*-value of the Kruskal–Wallis test reflects the change in the concentration index compared to the previous year, ^*^p < 0.05, ^**^p < 0.01, ^***^p < 0.001.

**Figure 3:**
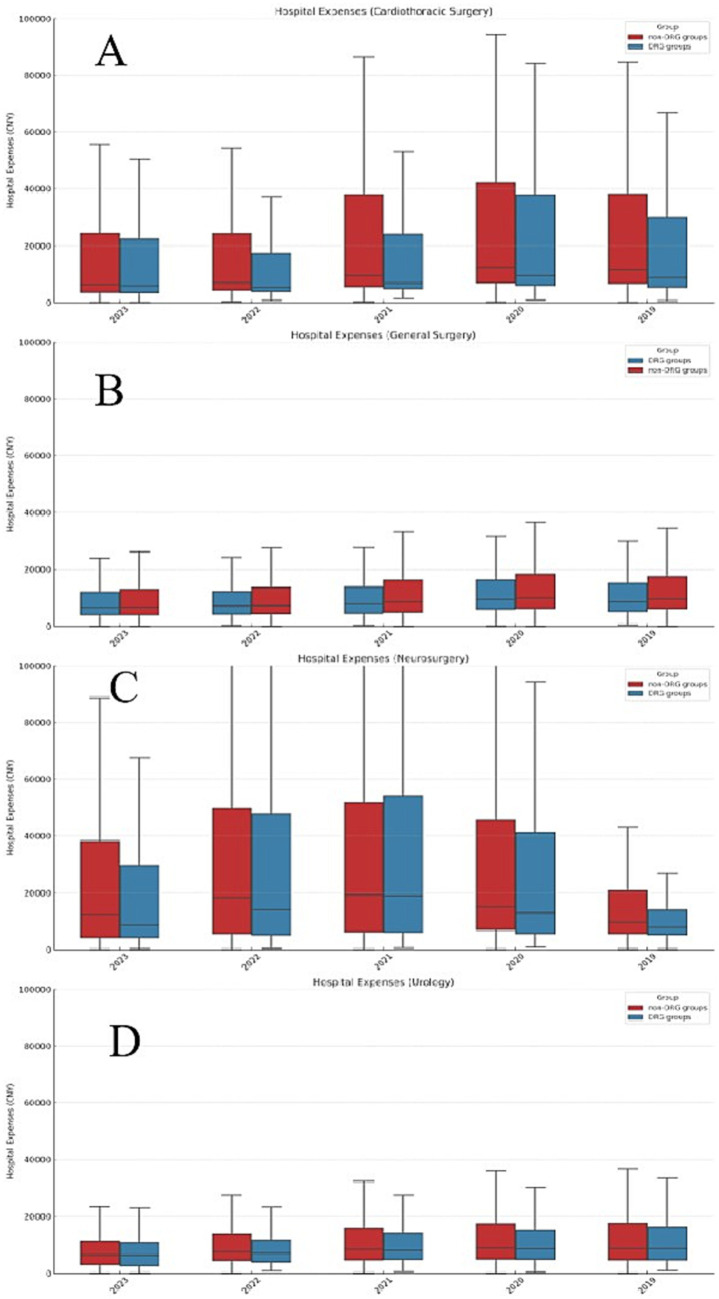
Box plot of hospitalization expenses by surgery type. **(A)** General surgery: The DRG payment group shows a more concentrated cost distribution, with a narrower interquartile range, indicating reduced cost variability. In contrast, the non-DRG group displays a wider range with more outliers, indicating greater cost dispersion. **(B)** Cardiothoracic surgery: The DRG payment group demonstrates a higher concentration of costs, suggesting better cost control and less variability. The non-DRG group exhibits a broader distribution with several high-cost outliers. **(C)** Neurosurgery: The DRG payment group displays a highly concentrated cost distribution, with a smaller interquartile range compared to the non-DRG group, where costs are more spread out, especially with the presence of high-cost outliers. **(D)** Urology: The DRG payment group’s cost distribution is more concentrated, indicating that the DRG system effectively reduces cost variability.

The distribution of inpatient costs and changes in cost concentration between the DRG payment group and the non-DRG payment group across different types of surgeries are shown in Figure 3. The distribution of inpatient costs in general surgery shows that the cost distribution in the DRG payment group is more concentrated compared to the non-DRG payment group. In cardiothoracic surgery, the cost distribution curve of the DRG payment group is notably tighter toward the center, concentrated within a narrower range of costs. In contrast, the non-DRG payment group shows a more dispersed cost distribution with a wider range of cost fluctuations. The distribution of inpatient costs in neurosurgery indicates that the DRG payment group has a significantly higher cost concentration compared to the non-DRG payment group. The cost distribution in the DRG payment group is more concentrated around the median, with a relatively narrow range, indicating more consistent costs among most patients. The cost distribution in urology surgery shows a higher concentration of costs in the DRG payment group. Compared to the non-DRG payment group, the cost distribution curve for the DRG payment group is steeper, concentrated within a narrower range of costs. Overall, the cost concentration of inpatient costs in the DRG payment group for all types of surgeries has gradually increased in the years following the reform, resulting in a more concentrated cost distribution.

### Readmission and complication rates in surgical departments pre- and post-DRG implementation

Readmission and complication rates before and after the implementation of DRG payment reform were compared across four major surgical departments (Supplementary Table 2). In the Cardiothoracic Surgery department, the mean readmission rate was 1.73% before DRG implementation and 1.69% after, with no statistically significant difference (*p* = 0.911). The corresponding complication rates were 0.59% and 0.87% (*p* = 0.308). In General Surgery, the readmission rate remained stable at 0.86% in both periods (*p* = 0.988), while the complication rate decreased from 0.46% to 0.24%, approaching statistical significance (*p* = 0.060). Neurosurgery showed an increase in readmission rates from 1.52% to 1.73% (*p* = 0.092) and a decrease in complication rates from 1.89% to 1.36% (*p* = 0.086). In the Urology department, the mean readmission rate increased from 2.45 to 3.08% (*p* = 0.108), and the complication rate rose from 0.11% to 0.19% (*p* = 0.056). No statistically significant differences were observed in any of the departments for either outcome.

### Comparison of hospitalization duration and material costs between DRG and non-DRG groups

As shown in Supplementary Table 3, both hospitalization days and material costs decreased across all four surgical departments after the implementation of DRG payment reform. In cardiothoracic surgery, the average length of stay declined from 11.3 to 9.79 days (*p* = 0.000), and material costs dropped from 6608.65 to 5415.15 CNY (*p* = 0.000). In urology, hospitalization decreased slightly from 8.77 to 8.49 days (*p* = 0.009), and material costs from 2399.06 to 2033.17 CNY (*p* = 0.000). General surgery showed a reduction in length of stay from 8.14 to 7.79 days (*p* = 0.000), and in material costs from 2835.71 to 2475.30 CNY (*p* = 0.000). In neurosurgery, hospitalization decreased from 15.75 to 14.37 days (*p* = 0.000), and material costs from 9070.75 to 8225.15 CNY (*p* = 0.020).

## Discussion

This study, through an analysis of inpatient costs between the DRG payment group and the non-DRG payment group across different types of surgeries, reveals the significant effectiveness of DRG payment reform in reducing cost disparities and enhancing the standardization of healthcare services. The results indicate that DRG payment effectively controlled inpatient costs across various types of surgeries, particularly demonstrating strong cost control in reducing drug and material expenses. The DRG payment system, through its standardized payment model, reduced cost disparities between different hospitals and among different patients. In general surgery, cardiothoracic surgery, and neurosurgery, the cost distribution in the DRG payment group was significantly more concentrated than in the non-DRG payment group, indicating improved standardization of healthcare services and more rational and regulated resource utilization. Additionally, an analysis of socioeconomic status (SES) was conducted to determine whether significant differences existed between the treatment and control groups. The results showed no significant differences in socioeconomic indicators such as income levels and educational background, suggesting that both groups were similar in terms of SES. This similarity helps to ensure that the observed differences in hospitalization costs are less likely to be influenced by SES disparities, thus strengthening the validity of the study’s conclusions regarding the impact of DRG payment reform. Since this study was conducted using data from a single tertiary hospital, generalizability to the national level may be restricted. Tertiary comprehensive hospitals generally represent higher standards in medical technology, resource allocation, and management; however, uneven distribution of medical resources and variations in policy implementation across different regions of China may result in different outcomes of DRG payment reform. This aspect has not been deeply explored in the current study. Future research should incorporate comparative analyses across regions and multiple healthcare institution levels to enhance generalizability.

The study also shows that the effectiveness of DRG payment varies across different types of surgeries. Specifically, the cost control effect of DRG payment is more pronounced in neurosurgery and urology surgeries, whereas it is relatively moderate in cardiothoracic surgeries. This difference may be related to several factors: first, the complexity of the surgery significantly impacts the effectiveness of DRG payment. High-complexity surgeries (such as neurosurgery) typically require more medical resources and longer hospital stays. The DRG payment system, through stringent cost control, significantly reduces cost fluctuations in high-complexity surgeries, making costs more concentrated and controllable. In contrast, the cost control effect is relatively less significant for lower-complexity surgeries. Second, different types of surgeries have varying demands for medical resources and usage patterns. For resource-intensive surgeries (such as cardiothoracic surgeries), the effectiveness of DRG payment may be influenced by the efficiency of resource allocation. If the hospital’s resource use is already efficient, the cost control effect of DRG payment may be limited (13). However, for surgeries where resource use is wasteful or excessive, DRG payment can significantly reduce costs through resource reallocation and optimized use. Third, the severity of the patient’s condition also directly affects the effectiveness of DRG payment. In patients with complex conditions and multiple comorbidities, the standardized cost model of the DRG payment system can effectively reduce cost uncertainty due to the complexity of the condition, thereby better controlling inpatient costs. However, for patients with milder conditions, the cost concentration effect may be less pronounced, as standardized payment has a relatively smaller impact on these patients. In summary, DRG payment reform has shown significant effectiveness in reducing inpatient cost disparities and enhancing the standardization of healthcare services. However, its effectiveness varies across different types of surgeries, mainly influenced by factors such as surgical complexity, resource usage patterns, and patient condition. Future policy-making should fully consider these factors and tailor the optimization of the DRG payment system accordingly, to better achieve the dual goals of cost control and improved healthcare quality.

In support of this finding, our data show that cardiothoracic surgery had the highest baseline material costs (6608.65 CNY in the non-DRG group) and a relatively moderate reduction after DRG reform (down to 5415.15 CNY), whereas neurosurgery, with a longer average hospitalization duration (15.75 days pre-DRG), demonstrated a greater reduction in both length of stay and material costs. Urology, typically involving standardized and minimally invasive procedures, also showed a strong cost control effect. These patterns indicate that differences in baseline surgical complexity and resource intensity likely contribute to the variation in DRG effectiveness across departments. While detailed data on reimbursement structures and surgical case weights were not available in this study, the observed disparities suggest that resource-heavy departments such as cardiothoracic surgery may face intrinsic cost constraints that limit the relative gains under standardized DRG payment.

This study provides a detailed analysis of the effects of DRG payment reform across different types of surgeries, and the results show that DRG payment reform has achieved significant success in controlling inpatient costs and enhancing the standardization of healthcare services. However, the outcomes vary across different types of surgeries and differ from the results observed in international studies. Internationally, particularly in developed countries such as the United States, Germany, and Australia, numerous studies have explored the impact of the DRG payment system on inpatient costs and healthcare quality (14, 15). These studies generally find that DRG payment can significantly reduce inpatient costs and improve resource utilization efficiency. For example, after the full implementation of DRG payment in Germany, the growth rate of inpatient costs was effectively controlled, and the quality and efficiency of hospital services improved (16). This study aligns with these international studies in general, indicating that the DRG payment system has a positive role in cost control and service standardization (17). However, this study also identified some differences from international research. For instance, studies in Germany and the United States have shown that DRG payment is particularly effective in controlling costs for high-complexity surgeries, such as cardiac and neurosurgical procedures (18). In contrast, in this study, while the cost control effect for neurosurgery was notable, the effect for cardiothoracic surgery was relatively moderate. This difference may stem from the varying healthcare systems between China and Western countries, particularly in resource allocation, hospital operational models, and patient management. The distribution and usage patterns of medical resources in China may have influenced the implementation effects of DRG payment, which is an area that warrants further exploration in future research.

Domestic studies have gradually evaluated the implementation effects of DRG payment reform in different regions and hospitals across China. For example, some studies have found that after implementing DRG payment, hospitals in major cities such as Beijing and Shanghai have significantly controlled inpatient costs, with a noticeable reduction in cost disparities (19, 20). These findings are consistent with the overall trend observed in this study, indicating that DRG payment reform has a good effect on cost control in China. However, this study further refines the analysis to different types of surgeries, revealing the differential effects of DRG payment across various surgical types. This approach differs from the broad analyses conducted in other domestic studies. Although previous research has noted that DRG payment demonstrates greater advantages in complex surgeries, this study is the first to systematically compare the specific effects of DRG payment across multiple surgical types, thereby providing more detailed and in-depth evidence. Additionally, domestic studies have highlighted that the effects of DRG payment differ between primary care hospitals and major urban hospitals (21). This study focuses on tertiary comprehensive hospitals, which have higher levels of resources and service capabilities within China’s healthcare system, making the implementation effects of DRG payment more prominent. This finding contrasts with the results from studies on primary care hospitals, suggesting that when promoting policies, it is important to consider the specific circumstances of different hospitals to achieve the optimal effect of DRG payment.

In comparing the results of domestic and international studies, this study’s findings are generally consistent with international research in terms of DRG payment’s effectiveness in cost control and standardization. However, there are some differences in the effects observed for specific types of surgeries. Domestic research also supports the positive role of DRG payment (22), but this study offers a more comprehensive and in-depth understanding by refining the analysis to specific surgical types. These differences highlight the uniqueness and complexity of the implementation of DRG payment in Western and Chinese healthcare systems. Future research should further explore the reasons behind these differences to optimize the application of the DRG payment system in China.

This study reveals that the effects of DRG payment reform vary significantly across different types of surgeries and levels of complexity. Therefore, policymakers should consider developing differentiated DRG payment policies that more accurately reflect the actual circumstances of various hospitals and the complexity of surgeries, in order to optimize resource allocation and enhance healthcare quality.

First, adjust payment standards based on hospital levels and surgical complexity. Tertiary hospitals, secondary hospitals, and primary healthcare institutions differ significantly in terms of resource allocation, patient population, and healthcare service levels. Therefore, DRG payment standards should be appropriately adjusted according to hospital levels. In tertiary general hospitals, especially those handling complex, high-risk surgeries, DRG payment policies should consider higher payment standards to cover the complex cases and resource-intensive treatment processes involved in these surgeries. Conversely, secondary hospitals and primary healthcare institutions can have lower payment standards to reflect the relatively simpler surgeries and low-complexity patient populations in these hospitals. This differentiated payment standard design can prevent hospitals from refusing complex cases due to concerns about reduced revenue, while also promoting the rational allocation of medical resources. For example, cardiothoracic surgery in tertiary hospitals may require higher payment standards to cover the high costs of equipment use and postoperative care, while secondary hospitals can set lower payment standards for relatively low-complexity surgeries, ensuring that patients receive necessary surgical treatment in primary healthcare settings.

Second, flexibly establish payment standards to balance cost control between complex and simple surgeries. One key challenge faced by the DRG payment system is how to achieve a balance in cost control between complex and simple surgeries. This study found differences in cost control effectiveness between high-complexity surgeries such as neurosurgery and cardiothoracic surgery. Therefore, policymakers should flexibly set payment standards based on the complexity and risk levels of surgeries. For high-complexity surgeries, higher payment standards should be set to encourage hospitals to accept and manage complex cases. For example, neurosurgery involves complex intraoperative monitoring and postoperative management, requiring hospitals to allocate more medical resources and skilled personnel. The payment standards should cover these additional costs to prevent difficulties in resource allocation due to excessively low payment standards. Conversely, for low-complexity surgeries, such as standardized procedures in general surgery and urology, payment standards should be optimized to reduce unnecessary high payments, thereby improving resource utilization efficiency while ensuring the quality of care.

Third, establish a dynamic adjustment mechanism to ensure the long-term effectiveness of payment standards. To maintain the long-term effectiveness of the DRG payment system, policymakers should establish a dynamic adjustment mechanism to regularly update payment standards and policies based on advances in medical technology, changes in surgical complexity, and hospital operations. It is recommended to establish a national or regional medical cost monitoring system to collect and analyze surgical cost data from hospitals in real-time. Using this data, policymakers can promptly identify discrepancies between payment standards and actual medical costs and make corresponding adjustments. Special attention should be given to surgeries with high growth rates or high variability to avoid payment standards lagging behind changes in actual costs. If the cost control effects of certain types of surgeries do not meet expectations, it may be necessary to reassess the payment standards or provide additional support and guidance to hospitals to ensure the sustained effectiveness of DRG payment reform. Future studies should expand data collection efforts to include multicenter designs encompassing secondary hospitals, rural healthcare facilities, and specialty hospitals. Additionally, performing regional variation analyses, such as urban–rural comparisons, can provide deeper insights into differential implementation effects of DRG payment reform, offering comprehensive and precise evidence to guide future healthcare policy formulation.

In addition to cost control and quality indicators, potential behavioral responses by hospitals under DRG payment systems merit further investigation. Specifically, practices such as upcoding—intentionally assigning cases to higher-paying DRG categories—and selective patient admission—avoiding complex or high-cost patients—may undermine the goals of payment reform. Although this study did not have access to patient-level clinical or coding audit data to directly assess such behaviors, the possibility of strategic coding or patient selection cannot be ruled out. Future research should incorporate detailed audits of DRG coding practices, monitor shifts in case severity and composition, and explore the use of linked clinical and administrative datasets to detect potential gaming behaviors. Recognizing and addressing these risks is essential to ensuring the integrity and effectiveness of DRG-based payment reform.

In addition to the cost analysis, this study incorporated clinical outcome indicators, including readmission rates and complication rates, to assess the potential impact of DRG payment reform on care quality. The results showed no significant increase in adverse outcomes in the DRG group compared to the non-DRG group across all four surgical departments. These findings suggest that DRG payment reform was effective in reducing inpatient costs without compromising patient safety or treatment quality. This reinforces the potential of DRG reform to achieve dual goals of cost containment and quality assurance, which is essential for sustainable health system improvement.

### Limitations

Although this study provides important insights into the effects of DRG payment reform, there are still some limitations that need to be addressed in future research. First, there is the limitation of using data from a single hospital. The data for this study were sourced from a tertiary general hospital in China, and while the hospital is representative in terms of resource allocation and healthcare quality, the findings may not fully apply to other types of hospitals or regions. Differences in management models, patient demographics, and resource utilization across hospitals may lead to varying effects of DRG payment reform. As a result, the generalizability and applicability of the findings may be limited. Second, there is the issue of uncontrolled potential variables. Although this study controlled for key variables such as age, gender, and Charlson Comorbidity Index (CCI), there may still be uncontrolled potential factors such as surgical complexity, physician skill level, and hospital management systems, which could influence the study’s results. Additionally, changes in the external environment during policy implementation (such as adjustments in health insurance policies or fluctuations in drug prices) could also impact the effects of DRG payment reform. Third, there is the limitation of short-term effect evaluation. This study primarily focused on the short-term effects of DRG payment reform, whereas the long-term effects of the DRG payment system, particularly in terms of healthcare quality, patient outcomes, and hospital financial status, require longer observation and analysis. Therefore, the current findings may not fully capture the long-term impacts of DRG payment reform. One significant limitation of this study is the reliance on data from a single tertiary comprehensive hospital, excluding data from other levels and regions. This limitation restricts the generalizability of our findings to other healthcare institutions nationally. Future studies should adopt multicenter designs, covering urban and rural regions and various levels of healthcare institutions, including secondary and specialty hospitals, to enhance external validity and the applicability of policy recommendations.

Based on the findings and limitations of this study, future research can develop in the following directions: First, expanding the sample range. Future research should include a broader sample range, covering more types of hospitals (such as secondary hospitals and specialty hospitals) and healthcare institutions in different regions, to assess the generalizability and effectiveness of DRG payment reform across various healthcare settings. A multicenter study design can more comprehensively validate the findings of this study and explore differences in DRG payment effects between hospitals, which will help enhance the generalizability and applicability of the research results. Second, exploring the impact on other types of healthcare services. Future research should further investigate the effects of DRG payment on other types of healthcare services, such as outpatient services and chronic disease management, particularly in terms of resource allocation and service quality. This will help provide a more comprehensive understanding of the impact of the DRG payment system on the entire healthcare system and offer broader evidence for further optimization of the payment system. Third, assessing the long-term effects of DRG payment reform. Future research should focus on the long-term effects of DRG payment reform, including its impact on healthcare quality, patient outcomes, and hospital financial status. This will help to comprehensively evaluate the overall effectiveness of the DRG payment system in achieving sustainable cost control and improving the standardization of healthcare services. It will also provide more robust evidence for policymakers to ensure that DRG payment reform can continue to have a positive impact in the future.

Additionally, this study did not adjust cost data across years to a constant price level. Although this is a common practice in economic analyses, such adjustment was not feasible due to the lack of surgery-specific price indices and the use of real-time hospital billing data. However, healthcare prices in China remained relatively stable during the study period, minimizing the risk of inflation bias.

## Conclusion

This study systematically analyzed the impact of DRG payment reform on inpatient costs across different types of surgeries. The results demonstrate that DRG payment effectively reduced inpatient costs and improved the standardization of healthcare services. In general surgery, cardiothoracic surgery, neurosurgery, and urology, costs in the DRG group were significantly lower than in the non-DRG group, particularly for drug and material expenses. DRG payment also increased cost concentration and reduced the proportion of high-cost cases, especially in complex and high-risk surgeries, indicating improved resource allocation and efficiency.

These findings support the feasibility and effectiveness of DRG reform in tertiary hospitals and provide evidence for further promoting DRG-based payment models. Future research should examine the long-term effects and expand validation to other hospital types and regions. Further exploration of differential impacts across surgeries is also needed to inform more targeted policy design.

Beyond the empirical results, DRG payment reform aligns with China’s broader shift toward value-based care by promoting transparent, standardized, and efficient service delivery. Its implementation in tertiary hospitals offers a foundation for expanding reforms to lower-level institutions, with strategies tailored to regional differences in resources and capacity. DRG payment is expected to play a central role in building a sustainable, high-quality healthcare system in China.

## Data Availability

The original contributions presented in the study are included in the article/Supplementary material, further inquiries can be directed to the corresponding author.

## Supplementary material

SUPPLEMENTARY FIGURE 1 Love plot: standardized differences before and after matching. This figure illustrates the standardized mean difference in the key variables before and after matching, with all the variables falling below 0.1, indicating a good balance between the treatment and control groups.
