# Supplementary figures and images for: The impact of DRG payment reform on inpatient costs for different surgery types: an empirical analysis based on Chinese tertiary hospitals
Source: Front Public Health. 2025 Jun 3;13:1563204. doi: 10.3389/fpubh.2025.1563204 (PMC12170532; doi:10.3389/fpubh.2025.1563204)

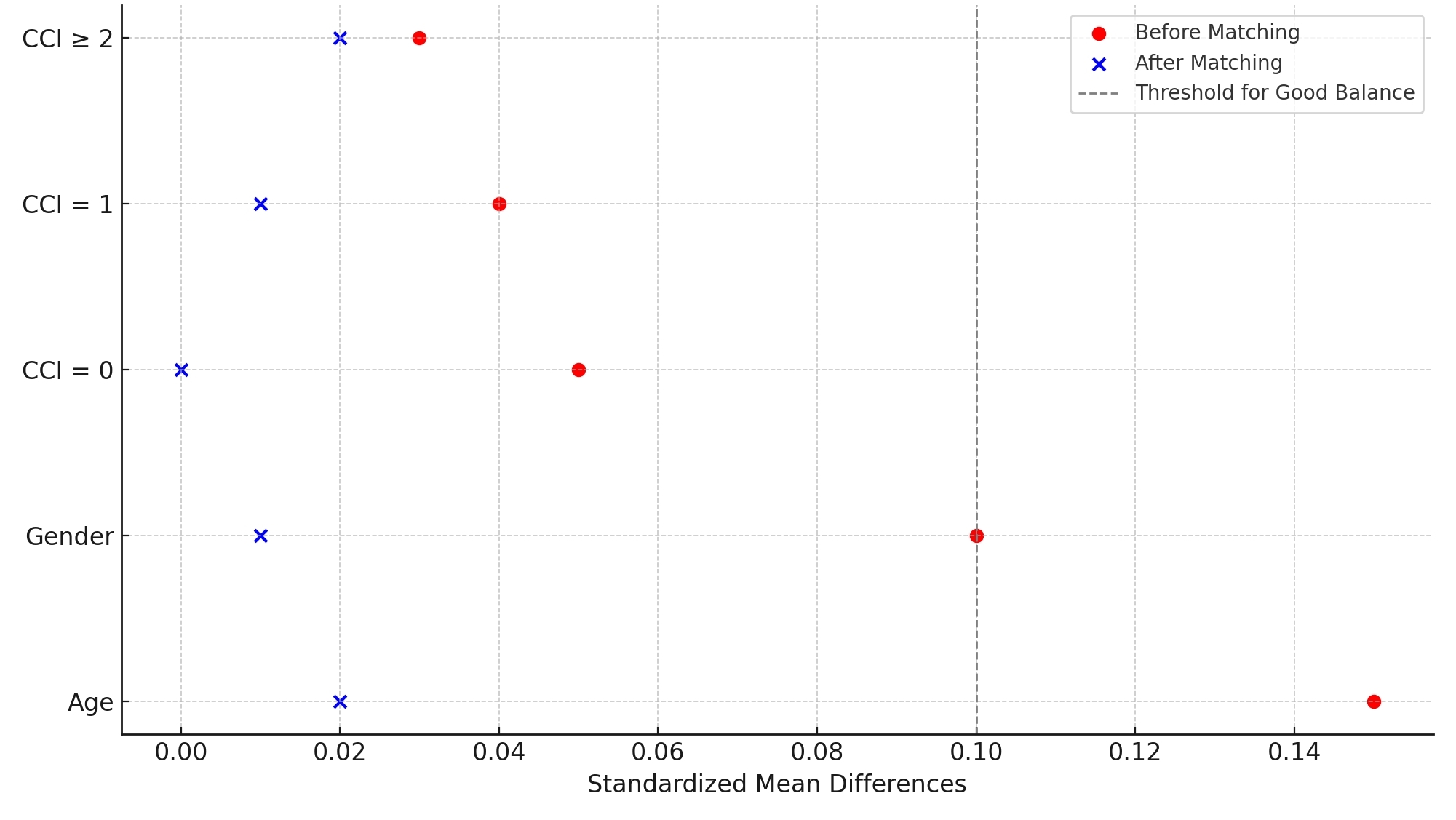

Supplement: SUPPLEMENTARY FIGURE 1 — Love plot: standardized differences before and after matching. This figure illustrates the standardized mean difference in the key variables before and after matching, with all the variables falling below 0.1, indicating a good balance between the treatment and control groups. [file Image_1.jpeg]
