# Supplementary material for: The impact of DRG payment reform on inpatient costs for different surgery types: an empirical analysis based on Chinese tertiary hospitals
Source: Front Public Health. 2025 Jun 3;13:1563204. doi: 10.3389/fpubh.2025.1563204 (PMC12170532; doi:10.3389/fpubh.2025.1563204)
Supplement: Supplementary file 3 [file Table_1.docx]

| **Supplementary Table 1. Representative Surgical Procedures by Department** | |
| --- | --- |
| **Department** | **Main Procedures** |
| **Urology** | Ureteroscopic holmium laser lithotripsy |
|  | Robot-assisted laparoscopic nephrectomy, pyeloplasty, or prostatectomy |
|  | Transurethral resection of the prostate using plasma technology |
|  | Ureteroscopic laser lithotripsy of the renal pelvis |
|  | Ureteroscopic holmium laser lithotripsy with stone extraction |
|  | Others |
| **General Surgery** | Minimally invasive vacuum-assisted breast lesion excision |
|  | Regional lymph node dissection |
|  | Laparoscopic appendectomy |
|  | Intraoperative neurophysiological monitoring |
|  | Autotransplantation of parathyroid gland |
|  | Others |
| **Neurosurgery** | Spinal instrumentation |
|  | Dural patch repair |
|  | Allogeneic bone grafting |
|  | Spinal fusion or refusion |
|  | Endovascular coiling for intracranial aneurysm |
|  | Others |
| **Cardiothoracic Surgery** | Thoracoscopic lobectomy |
|  | Thoracoscopic pleural adhesiolysis |
|  | Coronary artery bypass grafting (CABG) |
|  | Valve replacement or repair |
|  | Thoracoscopic mediastinal lymph node dissection |
|  | Correction of congenital heart defects under cardiopulmonary bypass |
|  | Others |
