# Supplementary material for: The impact of DRG payment reform on inpatient costs for different surgery types: an empirical analysis based on Chinese tertiary hospitals
Source: Front Public Health. 2025 Jun 3;13:1563204. doi: 10.3389/fpubh.2025.1563204 (PMC12170532; doi:10.3389/fpubh.2025.1563204)
Supplement: Supplementary file 4 [file Table_2.docx]

| **Supplementary Table 2. Comparison of Readmission and Complication Rates Before and After DRG Implementation in Four Surgical Departments** | | | | | | |
| --- | --- | --- | --- | --- | --- | --- |
| **Surgical Department** | **readmission rates** | | ***P*** | **complication rates** | | ***P*** |
|  | **Pre-DRG** | **Post-DRG** |  | **Pre-DRG** | **Post-DRG** |  |
| **Cardiothoracic Surgery** | 1.73% | 1.69% | 0.911 | 0.59% | 0.87% | 0.308 |
| **General Surgery** | 0.86% | 0.86% | 0.988 | 0.46% | 0.24% | 0.06 |
| **Neurosurgery** | 1.52% | 1.73% | 0.092 | 1.89% | 1.36% | 0.086 |
| **Urology** | 2.45% | 3.08% | 0.108 | 0.11% | 0.19% | 0.056 |
