# Supplementary material for: The impact of DRG payment reform on inpatient costs for different surgery types: an empirical analysis based on Chinese tertiary hospitals
Source: Front Public Health. 2025 Jun 3;13:1563204. doi: 10.3389/fpubh.2025.1563204 (PMC12170532; doi:10.3389/fpubh.2025.1563204)
Supplement: Supplementary file 5 [file Table_3.docx]

| **Supplementary Table 3. Comparison of Average Length of Stay and Material Costs Between Non-DRG and DRG Groups Across Four Surgical Departments** | | | | | | |
| --- | --- | --- | --- | --- | --- | --- |
| **Surgical Department** | **Hospitalization** | | **P** | **Material Cost** | | **P** |
|  | **non-DRG** | **DRG** |  | **non-DRG** | **DRG** |  |
| **Cardiothoracic Surgery** | 11.3 | 9.79 | 0 | 6608.65 | 5415.15 | 0 |
| **Urology** | 8.77 | 8.49 | 0.009 | 2399.06 | 2033.17 | 0 |
| **General Surgery** | 8.14 | 7.79 | 0 | 2835.71 | 2475.3 | 0 |
| **Neurosurgery** | 15.75 | 14.37 | 0 | 9070.75 | 8225.15 | 0.02 |
