# Supplementary material for: The impact of DRG payment reform on inpatient costs for different surgery types: an empirical analysis based on Chinese tertiary hospitals
Source: Front Public Health. 2025 Jun 3;13:1563204. doi: 10.3389/fpubh.2025.1563204 (PMC12170532; doi:10.3389/fpubh.2025.1563204)
Supplement: Supplementary file 6 [file Table_4.docx]

**Supplementary Table 4 The SD and double diference value of hospitalization expenses of patients (USD)**

| **Surgical Department** | **Year** | **Control Group (SD, USD)** | **Control Group Change** | **Intervention Group (SD, USD)** | **Intervention Group Change** | **Double-Difference Value (USD)** |
| --- | --- | --- | --- | --- | --- | --- |
| **Cardiothoracic Surgery** | 2019 | 3969.3 | 3381.85 |  |  |  |
|  | 2020 | 4358.7 | 3576.39 | 486.21 | 277.02 | -209.18 |
|  | 2021 | 4121.02 | 4195.11 | -199.74 | 649.85 | 849.59 |
|  | 2022 | 2653.42 | 2378.61 | -1385.57 | -1733 | -347.43 |
|  | 2023 | 2769.7 | 2439.83 | 143.67 | 85.77 | -57.9 |
| **General Surgery** | 2019 | 2239.53 | 2103.43 |  |  |  |
|  | 2020 | 2411.5 | 2105.42 | 226.59 | 53.29 | -173.31 |
|  | 2021 | 2243.27 | 1846.88 | -147.24 | -240.21 | -92.97 |
|  | 2022 | 2013.43 | 1705.47 | -185.19 | -104.64 | 80.55 |
|  | 2023 | 1861.91 | 1764.77 | -130.74 | 76.89 | 207.63 |
| **Neurosurgery** | 2019 | 4247.91 | 3762.95 |  |  |  |
|  | 2020 | 6388.54 | 5673.45 | 2244.23 | 2002.28 | -241.95 |
|  | 2021 | 6234.47 | 6144.6 | -98.46 | 520.53 | 618.99 |
|  | 2022 | 5549.89 | 5612.62 | -560.48 | -409.67 | 150.81 |
|  | 2023 | 4394.36 | 4411.4 | -1098.26 | -1143.3 | -45.04 |
| **Urology** | 2019 | 1653.19 | 1412.84 |  |  |  |
|  | 2020 | 1569.38 | 1664.61 | -43.49 | 286.23 | 329.72 |
|  | 2021 | 1849.61 | 1498.68 | 293.89 | -151.44 | -445.33 |
|  | 2022 | 1447.14 | 1284.19 | -365.65 | -184.66 | 180.99 |
|  | 2023 | 1574.9 | 1817.84 | 142.69 | 546.9 | 404.22 |
